# Radiomics and clinical data predict pseudoprogression after radiotherapy in high-grade glioma

**DOI:** 10.3389/fonc.2026.1892760

**Published:** 2026-07-20

**Authors:** Jiang Zhou, Zhang Danmeng, Yang Hui, Xu Zhuohua, Wei Mingjing, Lu Ying

**Affiliations:** 1 Department of Oncology, The Fourth Affiliated Hospital of Guangxi Medical University, Liuzhou, China; 2 Guangxi Key Laboratory of Intelligent Radiotherapy and Translational Research for Malignant Tumors, Liuzhou, China

**Keywords:** high-grade glioma, nomogram, prediction model, pseudoprogression, radiomics

## Abstract

**Background:**

On conventional magnetic resonance imaging, pseudoprogression after radiotherapy for high-grade glioma may closely resemble true tumor progression, leading to unnecessary surgery, premature treatment escalation, or delayed appropriate therapy. We developed and internally validated an exploratory multivariable model for individualized pseudoprogression risk estimation.

**Methods:**

This single-center retrospective cohort included 222 patients with World Health Organization 2021 central nervous system grade 3 or 4 glioma who underwent surgery followed by radiotherapy at Liuzhou Workers’ Hospital from January 2015 to December 2024. Pseudoprogression was adjudicated before modeling through multidisciplinary review; 13 pseudoprogression cases had histopathological confirmation, and non-histologically confirmed cases required at least 6 months of stable or improved follow-up imaging. The complete two-reader radiomics matrix was analyzed using a strict split-first workflow. Dataset partitioning preceded ICC filtering, Z-score normalization, and LASSO modeling.

**Results:**

A total of 3,404 radiomic features were analyzed. After reproducibility filtering and LASSO selection, 17 radiomic features were retained to construct the locked RadScore. In the held-out validation cohort, the integrated model combining RadScore, rCBV, ADC, NLR, MGMT promoter methylation, and TMZ treatment achieved an AUC of 0.811 (95% CI: 0.696-0.925). The clinical-imaging model without RadScore achieved a validation AUC of 0.744 (95% CI: 0.614-0.873), whereas RadScore alone achieved an AUC of 0.771 (95% CI: 0.648-0.894).

**Conclusion:**

The integrated model showed acceptable internal validation performance and provided a clinically interpretable framework for individualized PsP risk estimation by combining radiomic, perfusion-diffusion, inflammatory, molecular, and treatment-related information. External validation with standardized imaging protocols is warranted.

## Introduction

High-grade gliomas (HGGs) are the most common primary malignant tumors of the central nervous system and remain clinically aggressive, with marked invasiveness, biological heterogeneity, and poor prognosis (1). Standard treatment usually consists of maximal safe resection followed by radiotherapy with temozolomide (TMZ); however, survival remains limited despite this multimodal approach (2–4). After radiotherapy, treatment-related pseudoprogression (PsP) can be difficult to separate from true progression (TP) on conventional magnetic resonance imaging (MRI) (5).

PsP is generally regarded as a treatment-related imaging phenomenon driven by radiation-induced inflammation, increased vascular permeability, blood-brain barrier disruption, and focal necrosis rather than viable tumor growth. It may appear as new or enlarging enhancement within the irradiation field, often accompanied by edema, and is most often encountered during the first several months after radiotherapy. Reported incidence varies widely because studies differ in diagnostic criteria, treatment regimens, molecular subgroups, and follow-up duration (6–9). Misclassifying PsP as TP may lead to unnecessary surgery, inappropriate treatment escalation, or premature discontinuation of effective therapy; conversely, misclassifying TP as PsP may delay needed treatment (10).

Current assessment still relies heavily on Response Assessment in Neuro-Oncology (RANO)-based longitudinal follow-up, which requires serial imaging and can be influenced by reader judgment (11, 12). Diffusion-weighted and perfusion-weighted imaging provide additional biological information, but performance varies with acquisition protocols, post-processing, lesion timing, and region-of-interest definition (13, 14). Radiomics offers a complementary quantitative strategy by extracting high-dimensional descriptors of intensity, shape, and texture that are not fully captured by visual interpretation (15–18). Previous radiomics studies have reported encouraging discrimination between PsP and TP, yet imaging-only models may generalize poorly and may not capture the clinical, molecular, inflammatory, and treatment-related mechanisms underlying PsP (19–23).

In this context, we aimed to develop and internally validate a diagnostic multivariable model integrating MRI radiomics with clinical, molecular, treatment-related, diffusion, and perfusion variables to estimate the probability of PsP after radiotherapy in patients with HGG. Model performance was evaluated in terms of discrimination, calibration, and clinical utility using receiver operating characteristic analysis, calibration assessment, split-sample internal validation, and decision curve analysis. This article is reported in accordance with the TRIPOD checklist.

## Materials and methods

### Study design and participants

This retrospective prediction-model development and internal validation study was based on a consecutive cohort from Liuzhou Workers’ Hospital, a single tertiary center. We screened patients with pathologically confirmed World Health Organization 2021 central nervous system grade 3 or 4 glioma who underwent surgery followed by postoperative radiotherapy between January 2015 and December 2024. Because original pathology reports had been issued across different calendar years, all diagnoses and WHO grade assignments were re-reviewed according to the 2021 WHO Classification of Tumours of the Central Nervous System before analysis. The intended clinical application was risk estimation at the time of first suspected radiological progression after radiotherapy, when clinicians must decide whether new or enlarging enhancement is more likely to represent PsP or TP. Outcome follow-up was based on the availability of 1-year post-radiotherapy MRI or the last eligible MRI within the follow-up period.

Eligible patients had pathologically confirmed HGG, received postoperative radiotherapy, had postoperative MRI available, including T1-weighted, contrast-enhanced T1-weighted, T2-weighted, and T2-fluid-attenuated inversion recovery sequences, and had adequate follow-up imaging for PsP assessment. Patients were excluded if clinical, imaging, molecular, or outcome data required for model development were incomplete; if image quality was insufficient for segmentation or feature extraction; or if antitumor treatment had been administered before the index surgery or radiotherapy course.

Sex was abstracted from medical records and reported as female or male according to recorded biological sex. Race and ethnicity were not included because they were not routinely recorded in the source database and were not prespecified as clinically relevant for this single-center cohort. Predictor and outcome missingness was handled by complete-case analysis; no imputation was performed.

The study protocol, entitled “Construction of a Multimodal Prediction Model for Pseudoprogression After Radiotherapy in High-Grade Glioma”, was approved by the Medical Research Ethics Committee of Liuzhou Workers’ Hospital (approval No. KY2026255). The study was conducted in accordance with the Declaration of Helsinki and subsequent amendments. The requirement for individual informed consent was waived because of the retrospective design.

### Outcome definition

The target outcome was PsP after radiotherapy. PsP was diagnosed according to RANO/RANO 2.0-based longitudinal criteria: new or increased enhancement within the irradiation field after radiotherapy, no clear clinical deterioration attributable to tumor progression, and subsequent stability or improvement on follow-up imaging without treatment change. Thirteen PsP cases underwent repeat surgery or biopsy with histopathological confirmation. For patients without histopathological confirmation, a minimum of 6 months of stable or improved follow-up imaging after the suspicious progression event was required before classification as PsP. Outcome adjudication was performed through multidisciplinary review by two radiologists and two medical oncologists, and radiomic features were not used for outcome assignment. Cases with unresolved clinical-radiological evolution were excluded before formation of the final 222-patient analytic cohort; 21 indeterminate cases were excluded for this reason.

### Candidate predictors

Candidate predictors were prespecified on the basis of clinical relevance and prior literature. Collected variables included sex, age, Eastern Cooperative Oncology Group performance status, extent of resection, radiotherapy dose and technique, tumor volume, edema, tumor location, seizure at presentation, isocitrate dehydrogenase 1 (IDH1) mutation status, O6-methylguanine-DNA methyltransferase (MGMT) promoter methylation status, TMZ treatment, neutrophil-to-lymphocyte ratio (NLR), relative cerebral blood volume (rCBV), apparent diffusion coefficient (ADC), dexamethasone dose, and radiomics score (RadScore). MRI radiomic features, ADC, and rCBV were derived from the assessment performed at first suspected radiological progression after radiotherapy. NLR was calculated from peripheral neutrophil and lymphocyte counts obtained within 1 week before or after the corresponding MRI assessment. TMZ treatment was coded as positive if the patient received TMZ at any time during postoperative oncologic treatment. Dexamethasone dose was recorded as the cumulative dose during treatment. Molecular markers were obtained from postoperative pathology reports after 2021 WHO re-review.

Molecular marker assessment: Original pathology reports were issued across different years and were subsequently re-reviewed according to the 2021 WHO Classification of Tumors of the Central Nervous System before analysis. IDH1-R132H mutation status was assessed by immunohistochemistry (IHC). Positive IHC staining was defined as unequivocal brown-yellow cytoplasmic and/or nuclear staining in tumor cells; cases with staining in at least 10% of tumor cells were classified as IDH1-R132H mutant, whereas scattered staining confined to non-neoplastic cells such as infiltrating macrophages was considered negative. MGMT promoter methylation status was assessed using qualitative methylation-specific polymerase chain reaction (MSP). Cases were classified as MGMT methylation positive when the methylated-primer amplification band (M band) was clearly visible, or when its optical density or abundance was greater than or equal to that of the unmethylated band (U band).

### MRI acquisition and preprocessing

Radiomics analysis used postoperative follow-up MRI at the first suspected radiological progression after radiotherapy. MRI examinations were performed on two 3.0-T scanner platforms: a Siemens Healthineers MAGNETOM Skyra system equipped with a 32-channel head/neck coil and a GE Healthcare SIGNA Pioneer system equipped with a 32-channel high-resolution head coil. Conventional MRI included axial T1WI, axial T2WI, T2-FLAIR, diffusion-weighted imaging for ADC maps, and high-resolution contrast-enhanced 3D T1WI. DSC-PWI was acquired using gradient-echo echo-planar imaging. Detailed acquisition parameters are summarized in **Supplementary Table 1**. Scanner vendor and protocol variability were considered when interpreting the generalizability of radiomic, ADC, and rCBV measurements.

Images were converted from Digital Imaging and Communications in Medicine format to Neuroimaging Informatics Technology Initiative format. Skull stripping was performed with the Swiss Skull Stripper plugin in 3D Slicer (version available at the time of analysis; RRID: SCR_005619), and images were registered to the Montreal Neurological Institute 152 template using SlicerANTs. Bias-field correction used the N4 algorithm in SimpleITK with an Otsu-generated mask, 200 histogram bins, maximum iterations of 50, 50, 30, and 20 across four resolution levels, and a convergence threshold of 1e-6. Intensity normalization was performed using the WhiteStripe package in R on skull-stripped images after N4 correction, with the image-type parameter set according to MRI weighting. Images were then resampled to isotropic 1 x 1 x 1 mm voxels using SimpleITK B-spline interpolation before radiomic feature extraction (24–30).

### Region-of-interest delineation and radiomic feature extraction

Two radiologists independently delineated the enhancing tumor region of interest on contrast-enhanced T1-weighted imaging (T1CE). The T1CE-derived ROI was then registered to the corresponding T1-weighted, T2-weighted, and T2-fluid-attenuated inversion recovery sequences for multisequence radiomic feature extraction. Interobserver reproducibility was assessed by calculating intraclass correlation coefficients from features extracted from the two independent delineations. Discrepancies were resolved by consensus, and the final consensus ROI was used for model development. During delineation and consensus review, readers were not allowed to use radiomic features or model outputs for outcome assignment.

**Figure 1:**
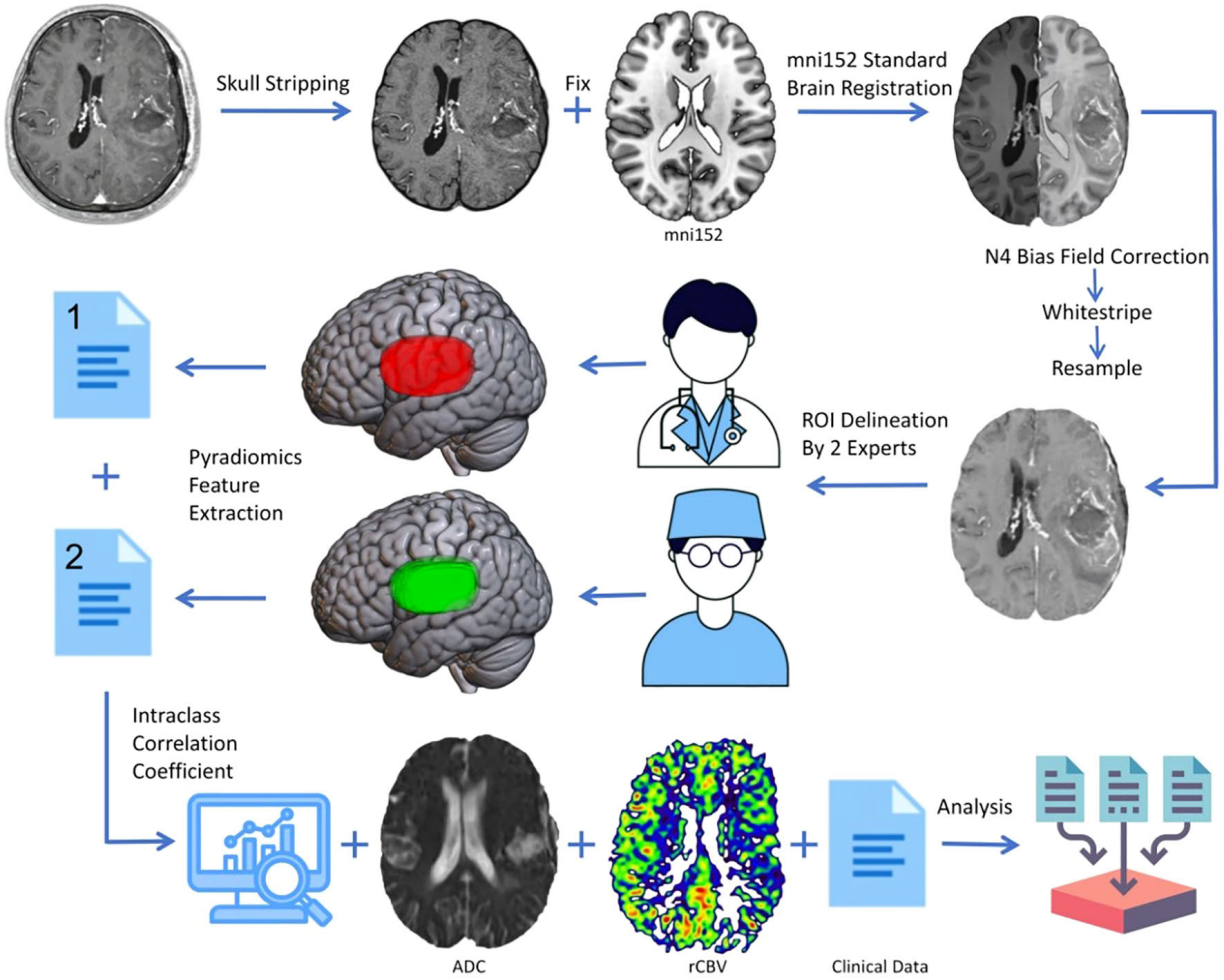
Radiomics workflow for extracting magnetic resonance imaging features in glioma. The workflow included image preprocessing, region-of-interest delineation, radiomic feature extraction, reproducibility assessment, and integration with clinical, molecular, diffusion, and perfusion variables. ADC, apparent diffusion coefficient; rCBV, relative cerebral blood volume; ROI, region of interest.

Radiomic features were extracted using PyRadiomics (version 3.0; **Figure 1**) from the T1CE-defined enhancing-tumor ROI registered to each sequence. The extraction settings were derived from the original feature-extraction script: fixed bin width was set to 25, the ROI label value was 1, resampledPixelSpacing was set to None, and interpolateResampling was set to False because images had already been resampled before feature extraction. Original and Wavelet image types were enabled; diagnostic metadata returned by PyRadiomics was removed before analysis. Shape, first-order intensity, and higher-order texture features, including gray-level co-occurrence matrix, gray-level run-length matrix, gray-level size zone matrix, gray-level dependence matrix, and neighboring gray-tone difference matrix features, were extracted.

### Feature selection and RadScore construction

**Figure 2:**
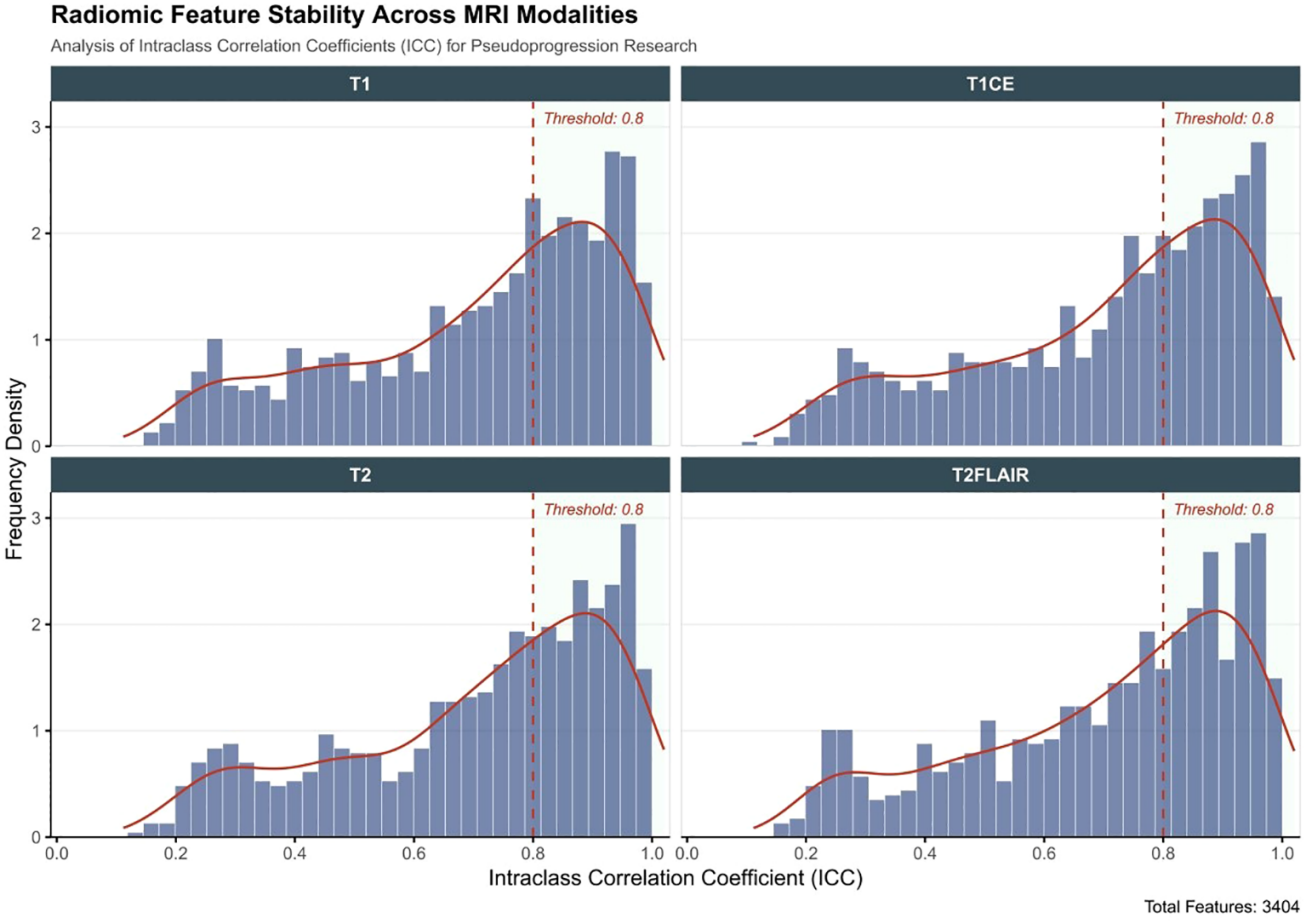
Intraclass correlation coefficient analysis for radiomic feature stability across magnetic resonance imaging modalities. Features with intraclass correlation coefficients greater than 0.80 were retained for downstream modeling.

Radiomic features from the four MRI sequences were analyzed using a strict split-first workflow to prevent overfitting and information leakage. The dataset was first partitioned into training and validation cohorts in a stratified 7:3 ratio. All subsequent radiomics steps were restricted to the training cohort: interobserver reproducibility was assessed using ICCs calculated from the two independent reader matrices, stable features were retained using an ICC threshold of 0.80 or greater (**Figure 2**), Z-score normalization parameters were derived only from the training cohort, and LASSO logistic regression with 10-fold cross-validation was fitted only in the training cohort. The locked stable-feature list, training-derived means and standard deviations, LASSO-selected features, and LASSO coefficients were then applied unchanged to the validation cohort.

### ADC and rCBV measurement

ADC was calculated from diffusion-weighted imaging acquired with b values of 0 and 1,000 s/mm2. ADC maps were generated with the exponential decay model S(b) = S (0) x exp(-b x ADC), and mean ADC was measured within the tumor-enhancement region of interest at the first suspected radiological progression after radiotherapy and expressed as x10^-3 mm2/s (31). During ADC measurement, regions of necrosis, hemorrhage, and large vessels were avoided whenever identifiable.

rCBV was derived from DSC-PWI obtained at the first suspected radiological progression after radiotherapy. DSC-PWI consisted of 60 dynamic phases with a temporal resolution of approximately 1.5 seconds. A preload dose of 0.05 mmol/kg gadolinium-based contrast agent was administered approximately 5 minutes before perfusion imaging to reduce T1-leakage effects. During dynamic acquisition, a main bolus dose of 0.1 mmol/kg gadolinium-based contrast agent was injected at approximately the 10th phase using a power injector at 4.5 mL/s, followed by a 20-mL saline flush. Post-processing was performed on Syngo.Via or GE AW workstations using singular value decomposition, and bidirectional T1 and T2* leakage correction based on the Boxerman-Schmainda model was applied to calculate corrected rCBV. rCBV was defined as cerebral blood volume in the tumor region of interest divided by cerebral blood volume in contralateral normal-appearing white matter (32). Regions of necrosis, hemorrhage, and large vessels were avoided during ROI placement.

### Model development and statistical analysis

Continuous variables were summarized as median [interquartile range], and categorical variables were summarized as number (percentage). Between-group comparisons used the independent t test or Mann-Whitney U test for continuous variables, as appropriate, and the chi-square test or Fisher exact test for categorical variables. Two-sided P values less than 0.05 were considered statistically significant.

Continuous predictors were standardized before regression modeling unless a clinically interpretable scale was retained, and categorical predictors were entered as indicator variables. Univariable logistic regression was used to screen candidate predictors. Variables with P < 0.05 in univariable analysis were then entered into multivariable logistic regression to identify independent predictors and construct the final model. A nomogram was derived from the multivariable model using the rms package in R.

Model performance was evaluated across three domains: discrimination, calibration, and clinical utility. Discrimination was quantified using receiver operating characteristic curves and area under the curve with 95% confidence intervals. Calibration was assessed with calibration plots, calibration-in-the-large, calibration slope, concordance index, and Brier score. Clinical utility was examined using decision curve analysis. For the leakage-controlled nested analysis, a stratified split was performed with a fixed seed (2026): 70% of patients were assigned to a training cohort and 30% to a held-out validation cohort, preserving the proportion of PsP events. ICC filtering, Z-score normalization, LASSO feature selection, RadScore construction, and integrated-model coefficient estimation were all performed exclusively in the training cohort. The locked workflow was then applied to the validation cohort without feature reselection, parameter updating, or recalibration. Validation-set performance was compared across the integrated model, a RadScore-only model, and a clinical-imaging model that excluded RadScore and retained rCBV, ADC, NLR, MGMT promoter methylation status, and TMZ treatment.

Study size was determined by the number of eligible patients in the retrospective cohort. The full analytic cohort included 222 patients and 60 PsP events. In the split-sample analysis, the training cohort included 155 patients with 42 PsP events, and the validation cohort included 67 patients with 18 PsP events. Because the final multivariable model included six predictors, the split-sample analysis was treated as supportive internal validation, given the reduced statistical efficiency of data splitting in modest-sized datasets.

## Results

### Patient flow and baseline characteristics

**Figure 3:**
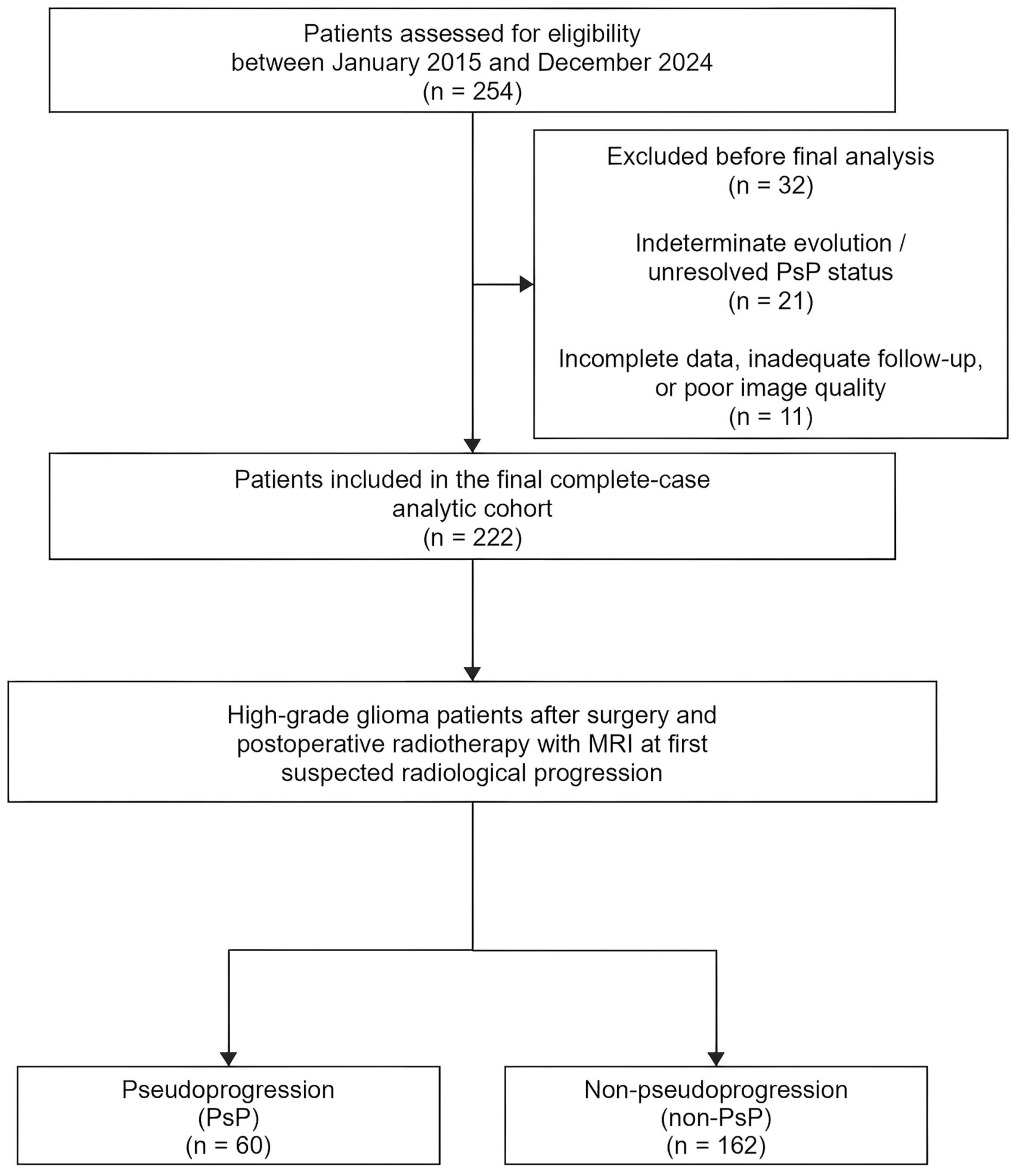
Flowchart of patient selection. A total of 254 patients were assessed for eligibility. Thirty-two patients were excluded before final analysis, including 21 patients with indeterminate clinical-radiological evolution or unresolved PsP/non-PsP status and 11 patients with incomplete required data, inadequate follow-up, or poor image quality. The final complete-case analytic cohort included 222 patients, comprising 60 patients with pseudoprogression and 162 patients without pseudoprogression.

Among 254 screened patients, 32 were excluded because of incomplete required data, inadequate follow-up information, insufficient image quality, or indeterminate post-treatment clinical-radiological evolution. The final complete-case analytic cohort included 222 patients, of whom 60 (27.0%) had PsP and 162 (73.0%) were classified as non-PsP. Among excluded patients, 21 had unresolved post-treatment evolution and were excluded before model development. After complete-case exclusion, no missing values remained for candidate predictors or the outcome. The patient selection process is shown in **Figure 3**.

Baseline characteristics according to PsP status are summarized in **Table 1**. The groups differed significantly in seizure at presentation, isocitrate dehydrogenase 1 status, MGMT promoter methylation status, TMZ treatment, NLR, rCBV, and ADC.

**Table 1:** Baseline characteristics of patients stratified by pseudoprogression status.

Variable	Category	Non-PsP (n=162)	PsP (n=60)	P value
Sex	Female	70 (43.2%)	23 (38.3%)	0.616
	Male	92 (56.8%)	37 (61.7%)	
Age (years)		48.0 [35.0, 59.8]	49.0 [38.0, 56.8]	0.678
ECOG performance status	0	52 (32.1%)	13 (21.7%)	0.314
	1	69 (42.6%)	30 (50.0%)	
	2	41 (25.3%)	17 (28.3%)	
Extent of resection	Biopsy	6 (3.7%)	2 (3.3%)	0.426
	Gross total resection	12 (7.4%)	8 (13.3%)	
	Partial resection	44 (27.2%)	19 (31.7%)	
	Subtotal resection	100 (61.7%)	31 (51.7%)	
RT dose (Gy)		60.2 [54.0, 64.3]	59.8 [54.0, 64.1]	0.985
RT technique	Gamma Knife	26 (16.0%)	12 (20.0%)	0.526
	IMRT	35 (21.6%)	14 (23.3%)	
	TOMO	38 (23.5%)	17 (28.3%)	
	VMAT	63 (38.9%)	17 (28.3%)	
Tumor volume (cm3)		86.0 [47.3, 165.2]	91.2 [45.9, 174.6]	0.888
Edema	Indeterminate	33 (20.4%)	11 (18.3%)	0.866
	No	91 (56.2%)	33 (55.0%)	
	Yes	38 (23.5%)	16 (26.7%)	
Location	Cortical	101 (62.3%)	39 (65.0%)	0.807
	Deep	29 (17.9%)	8 (13.3%)	
	Midline/ventricular	12 (7.4%)	6 (10.0%)	
	Posterior/brainstem	20 (12.3%)	7 (11.7%)	
Seizure	Yes	34 (21.0%)	21 (35.0%)	0.049
	No	128 (79.0%)	39 (65.0%)	
IDH1	Mutant	100 (61.7%)	27 (45.0%)	0.037
	Wild-type	62 (38.3%)	33 (55.0%)	
MGMT promoter	Unmethylated/negative	51 (31.5%)	6 (10.0%)	0.002
	Methylated/positive	111 (68.5%)	54 (90.0%)	
TMZ	Yes	53 (32.7%)	34 (56.7%)	0.002
	No	109 (67.3%)	26 (43.3%)	
NLR		2.3 [1.4, 2.9]	3.3 [2.2, 3.8]	<0.001
rCBV		2.1 [1.2, 2.8]	2.8 [2.0, 3.3]	<0.001
ADC		1.4 [1.1, 1.6]	1.0 [0.9, 1.3]	<0.001
Dexamethasone dose (mg)		32.5 [20.0, 43.8]	35.0 [25.0, 50.0]	0.241

Data are presented as median [interquartile range] for continuous variables and number (percentage) for categorical variables. ADC, apparent diffusion coefficient; ECOG, Eastern Cooperative Oncology Group; IDH1, isocitrate dehydrogenase 1; IMRT, intensity-modulated radiotherapy; MGMT, O6-methylguanine-DNA methyltransferase; NLR, neutrophil-to-lymphocyte ratio; PsP, pseudoprogression; rCBV, relative cerebral blood volume; RT, radiotherapy; TMZ, temozolomide; TOMO, tomotherapy; VMAT, volumetric modulated arc therapy.

### Radiomic feature selection and RadScore performance

**Figure 4:**
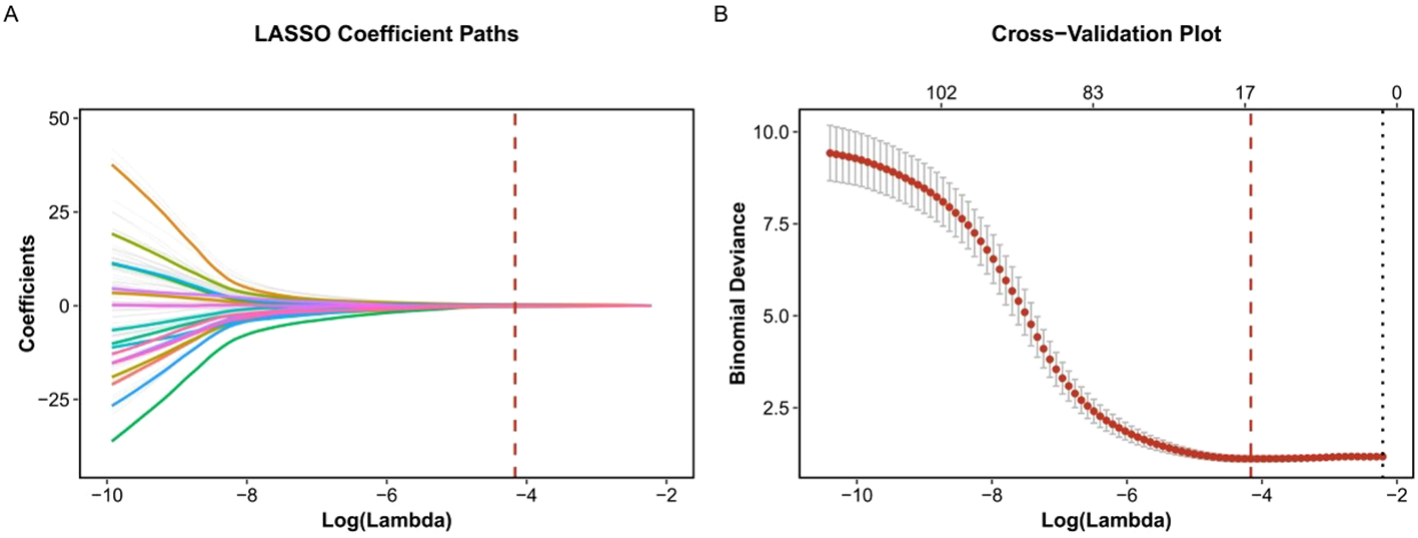
Radiomic feature selection using least absolute shrinkage and selection operator regression. **(A)** Coefficient path plot. **(B)** Cross-validation error plot used to select the optimal regularization parameter.

**Figure 5:**
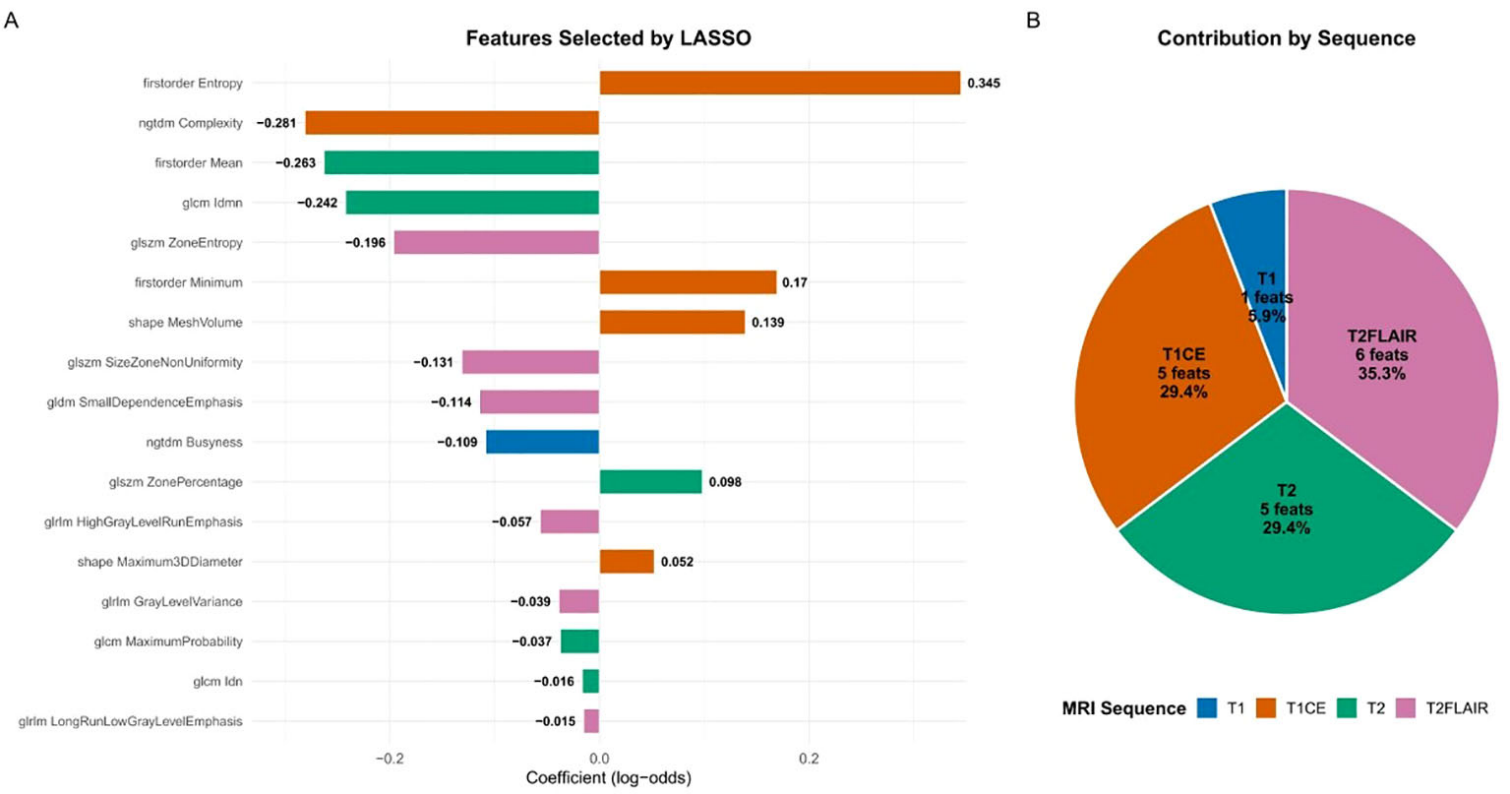
Contribution of selected radiomic features. **(A)** Bar plot of absolute least absolute shrinkage and selection operator coefficients for the 17 retained features. **(B)** Pie chart showing the relative contribution of magnetic resonance imaging sequences to the radiomics score.

For each patient, 3,404 radiomic features were analyzed from the four MRI sequences, representing 851 features from each of the T1WI, T1CE, T2WI, and T2-FLAIR sequences. Training-only ICC filtering retained 132 stable features. After training-set Z-score normalization, LASSO logistic regression with 10-fold cross-validation selected 17 non-zero-coefficient features (**Figure 4**): T1CE_original_firstorder_Entropy, T1CE_original_ngtdm_Complexity, T2_original_firstorder_Mean, T2_original_glcm_Idmn, T2FLAIR_original_glszm_ZoneEntropy, T1CE_original_firstorder_Minimum, T1CE_original_shape_MeshVolume, T2FLAIR_original_glszm_SizeZoneNonUniformity, T2FLAIR_original_gldm_SmallDependenceEmphasis, T1_original_ngtdm_Busyness, T2_original_glszm_ZonePercentage, T2FLAIR_original_glrlm_HighGrayLevelRunEmphasis, T1CE_original_shape_Maximum3DDiameter, T2FLAIR_original_glrlm_GrayLevelVariance, T2_original_glcm_MaximumProbability, T2_original_glcm_Idn, and T2FLAIR_original_glrlm_LongRunLowGrayLevelEmphasis. Their coefficients are shown in **Figure 5**.

**Figure 6:**
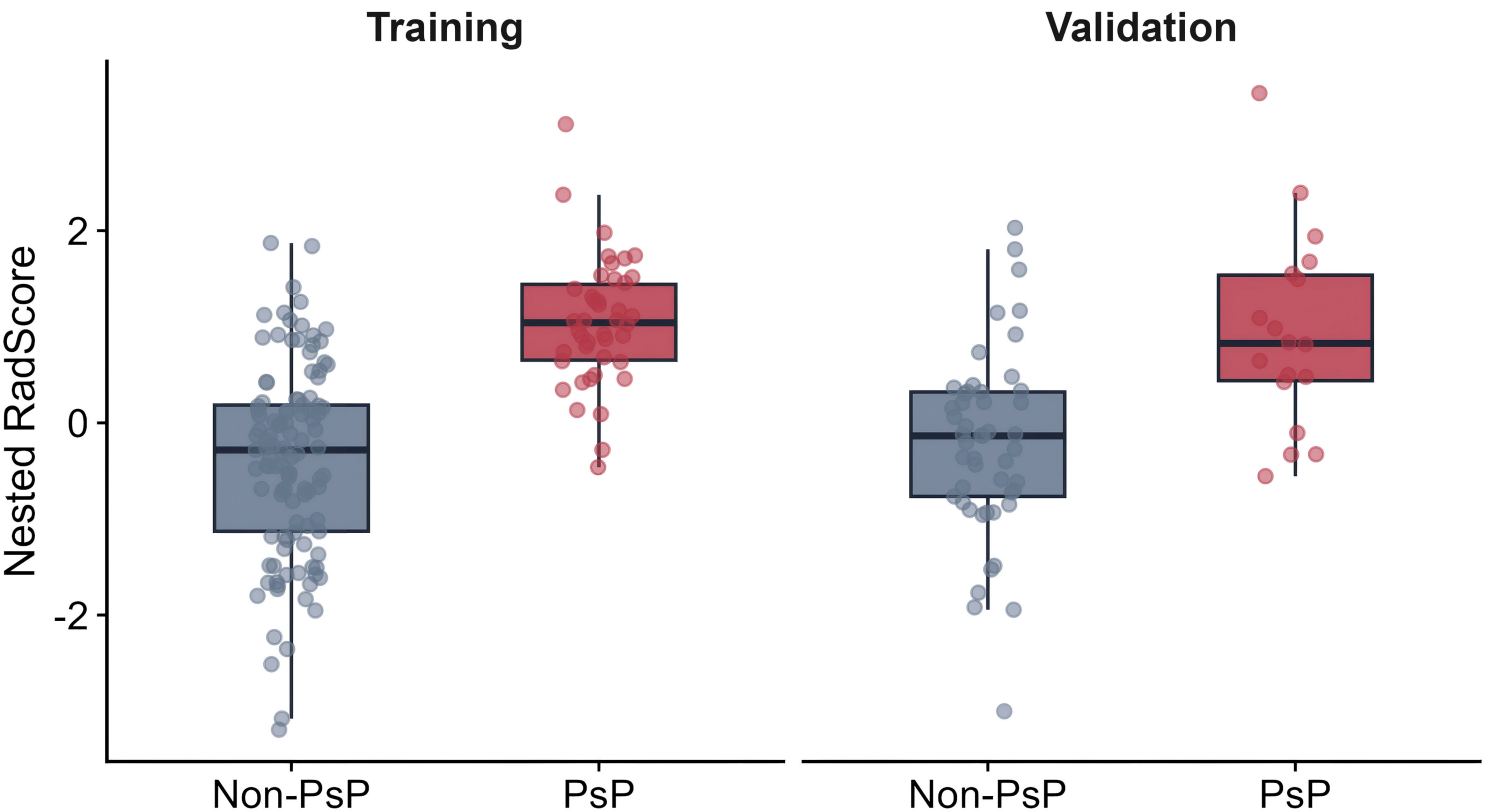
Distribution of the locked nested RadScore in the training and validation cohorts stratified by pseudoprogression status. Boxplots with overlaid individual points show RadScore values for non-PsP and PsP patients in each cohort. PsP, pseudoprogression.

In the leakage-controlled split-first analysis, the RadScore-alone model showed moderate and relatively stable discrimination. Its AUC was 0.890 (95% CI: 0.838-0.942) in the training cohort and 0.771 (95% CI: 0.648-0.894) in the validation cohort, suggesting that the nested radiomics signature retained predictive information after leakage-controlled internal validation. The distribution of the locked nested RadScore according to PsP status in the training and validation cohorts is shown in **Figure 6**.

### Clinical predictors and final model specification

**Figure 7:**
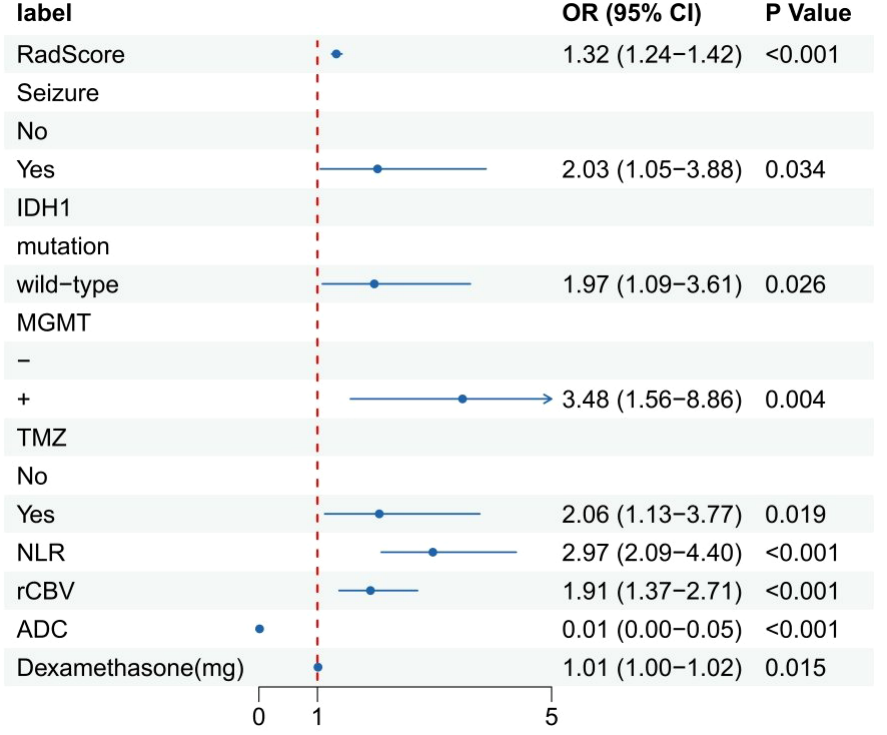
Forest plot of univariable logistic regression analysis for candidate predictors of pseudoprogression. CI, confidence interval; OR, odds ratio.

In univariable logistic regression, RadScore, seizure at presentation, isocitrate dehydrogenase 1 status, MGMT promoter methylation, TMZ treatment, NLR, rCBV, ADC, and dexamethasone dose were associated with PsP (all P < 0.05; **Figure 7**).

**Figure 8:**
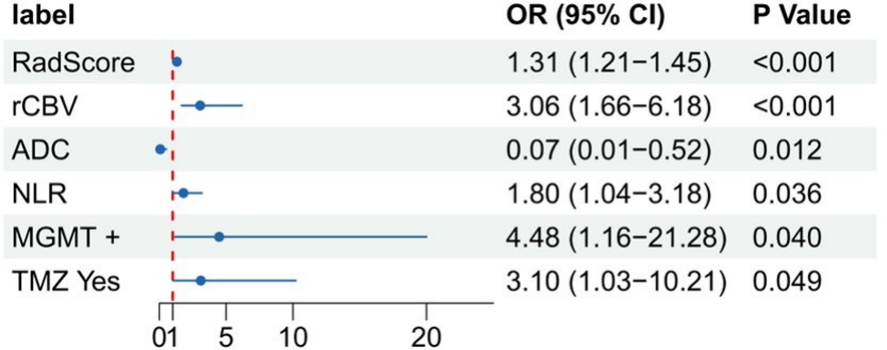
Forest plot of multivariable logistic regression analysis for independent predictors of pseudoprogression. CI, confidence interval; OR, odds ratio.

Multivariable logistic regression retained RadScore, rCBV, ADC, NLR, MGMT promoter methylation, and TMZ treatment as independent predictors of PsP. The adjusted results were RadScore (odds ratio, 1.31; 95% confidence interval: 1.21-1.45; P < 0.001), rCBV (odds ratio, 3.06; 95% confidence interval: 1.66-6.18; P < 0.001), ADC (odds ratio, 0.07; 95% confidence interval: 0.01-0.52; P = 0.012), NLR (odds ratio, 1.80; 95% confidence interval: 1.04-3.18; P = 0.036), MGMT promoter methylation positive status (odds ratio, 4.48; 95% confidence interval: 1.16-21.28; P = 0.040), and TMZ treatment (odds ratio, 3.10; 95% confidence interval: 1.03-10.21; P = 0.049; **Figure 8**).

**Figure 9:**
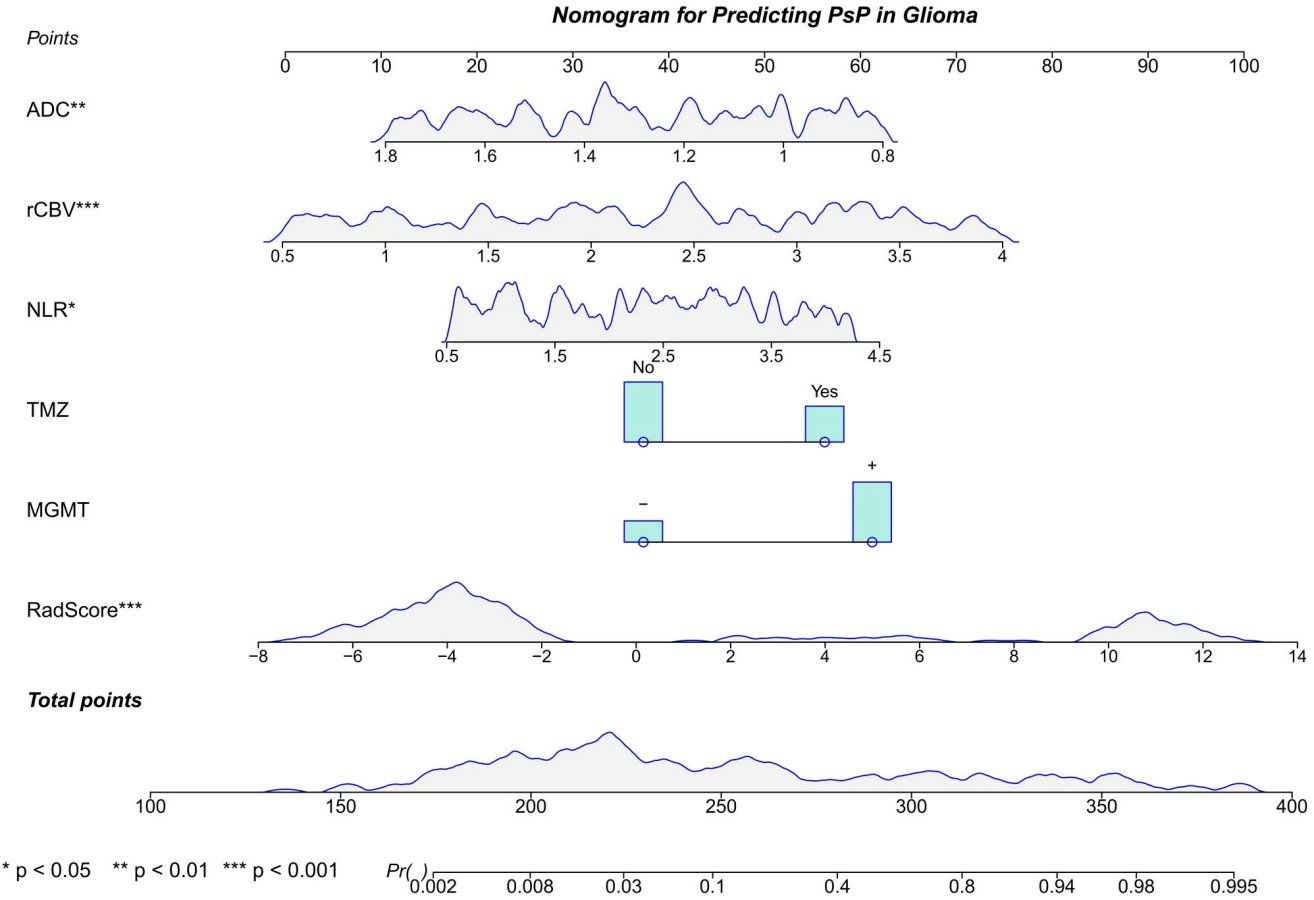
Nomogram for individualized prediction of pseudoprogression after radiotherapy in patients with high-grade glioma.

The final logistic regression equation was Logit(PsP) = -3.971 + 0.269 x RadScore + 1.174 x rCBV - 2.838 x ADC + 0.617 x NLR + 1.305 x MGMT(+) + 1.034 x TMZ(Yes). The predicted probability was calculated as P(PsP) = 1/[1 + exp(-Logit(PsP))]. MGMT(+) was coded as 1 for positive MGMT promoter methylation and 0 for negative methylation; TMZ(Yes) was coded as 1 for receipt of TMZ treatment and 0 for no TMZ treatment. The corresponding nomogram is shown in **Figure 9**.

### Model performance

**Figure 10:**
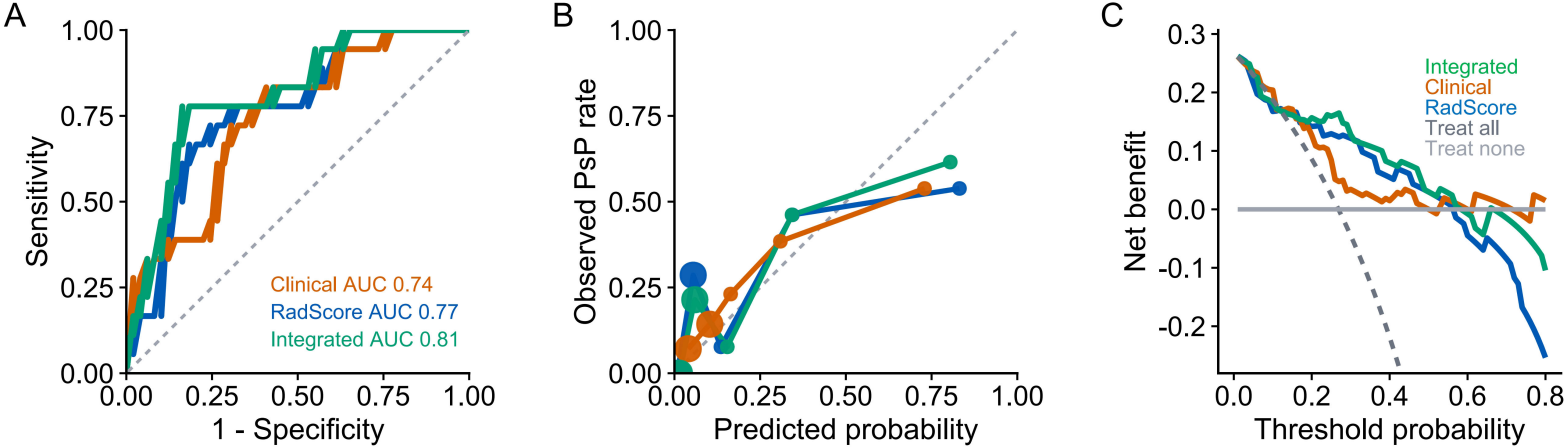
Split-sample internal validation and model comparison. **(A)** Receiver operating characteristic curves in the held-out validation cohort comparing the integrated model, RadScore-only model, and clinical-imaging model. **(B)** Calibration plot showing agreement between predicted and observed pseudoprogression probabilities. **(C)** Decision curve analysis comparing the integrated model, RadScore-only model, clinical-imaging model, treat-all strategy, and treat-none strategy. AUC, area under the receiver operating characteristic curve; PsP, pseudoprogression.

In the leakage-controlled split-first analysis, the integrated model showed an AUC of 0.898 (95% CI: 0.850-0.946) and a Brier score of 0.116 in the training cohort. In the held-out validation cohort, the integrated model achieved an AUC of 0.811 (95% CI: 0.696-0.925), a Brier score of 0.159, a mean predicted versus observed PsP rate of 0.268 versus 0.269, a calibration intercept of 0.006, and a calibration slope of 0.575. The clinical-imaging model without RadScore achieved a validation AUC of 0.744 (95% CI: 0.614-0.873) and a Brier score of 0.178, whereas RadScore alone achieved a validation AUC of 0.771 (95% CI: 0.648-0.894) and a Brier score of 0.185. Detailed performance metrics for the integrated, clinical-imaging, and RadScore-only models in the training and held-out validation cohorts are summarized in **Table 2**. The validation ROC curves, calibration plots, and decision curve analysis for the integrated model, clinical-imaging model, and RadScore-only model are shown in **Figure 10**.

**Table 2:** Performance of the integrated, clinical-imaging, and RadScore-only models in the stratified 7:3 split-sample internal validation analysis.

Model	Cohort	n/events	AUC (95% CI)	Brier score	Mean predicted/observed	Calib. intercept	Calib. slope
Integrated	Training	155/42	0.898 (0.850-0.946)	0.116	0.271 vs 0.271	0.000	1.000
Integrated	Validation	67/18	0.811 (0.696-0.925)	0.159	0.268 vs 0.269	0.006	0.575
Clinical-imaging	Training	155/42	0.863 (0.804-0.923)	0.133	0.271 vs 0.271	-0.000	1.000
Clinical-imaging	Validation	67/18	0.744 (0.614-0.873)	0.178	0.263 vs 0.269	0.043	0.589
RadScore only	Training	155/42	0.890 (0.838-0.942)	0.118	0.271 vs 0.271	0.000	1.000
RadScore only	Validation	67/18	0.771 (0.648-0.894)	0.185	0.268 vs 0.269	0.011	0.411

AUC, area under the receiver operating characteristic curve; CI, confidence interval. The leakage-controlled split-first workflow used 3,404 candidate radiomic features from complete two-reader matrices. Dataset partitioning preceded ICC filtering, Z-score normalization, and LASSO feature selection. The locked RadScore was constructed from LASSO-selected radiomic features. The integrated model included RadScore, rCBV, ADC, NLR, MGMT promoter methylation status, and temozolomide treatment; the clinical-imaging model excluded RadScore. Each model was fitted in the training cohort and applied to the validation cohort without recalibration.

## Discussion

This study developed and internally validated a multimodal prediction model for PsP after radiotherapy in patients with HGG. The final model integrated radiomic, perfusion, diffusion, inflammatory, molecular, and treatment-related information. RadScore, rCBV, ADC, NLR, MGMT promoter methylation, and TMZ treatment were independently associated with PsP. The integrated model showed high apparent discrimination, acceptable calibration, and favorable decision-curve net benefit in the development cohort, with maintained discrimination in the held-out internal validation cohort.

Most of the 17 radiomic features selected by least absolute shrinkage and selection operator regression were texture features, particularly from contrast-enhanced T1-weighted and T2-fluid-attenuated inversion recovery sequences. T1CE_original_firstorder_Entropy was the strongest positive contributor to RadScore, suggesting that signal disorder within enhancing tissue may reflect post-radiotherapy inflammation, vascular permeability, necrosis, and tissue disruption (33–37). Features capturing spatial complexity, zone entropy, and neighborhood gray-tone variation may further quantify subtle tissue patterns that are difficult to appreciate visually.

The independent associations of perfusion and diffusion parameters with PsP underscore the biological complexity of post-treatment enhancement. In many studies, TP is associated with neovascularization and higher perfusion, whereas PsP tends to show lower perfusion. The positive association between rCBV and PsP in this cohort should therefore be interpreted cautiously. One possible explanation is that early treatment-related injury may produce inflammatory hyperemia, severe blood-brain barrier disruption, and reactive permeability changes that can influence apparent rCBV estimates even when preload dosing and leakage correction are applied. Differences in imaging timing, ROI placement, scanner platform, and normalization strategy may also contribute to this observation (6, 38). The association between lower ADC and PsP should likewise be interpreted as hypothesis-generating; lower ADC may reflect transient hypercellularity related to inflammatory infiltration, cytotoxic edema, vascular injury, necrosis, or temporal variation in diffusion characteristics after treatment rather than viable tumor alone.

NLR was independently associated with PsP, supporting a potential contribution of systemic inflammation to post-radiotherapy imaging changes. Elevated NLR may reflect a pro-inflammatory state and altered immune balance, both of which could plausibly contribute to radiation-related inflammatory responses (39). MGMT promoter methylation and TMZ treatment were also independent predictors. MGMT promoter methylation is associated with sensitivity to alkylating therapy and has repeatedly been linked to higher rates of PsP, whereas TMZ combined with radiotherapy may intensify treatment-related injury and inflammatory response (40, 41). Recent methodological work on brain tumor segmentation, radiomics reproducibility, ICC reliability, penalized regression, clinical predictors, and calibrated radiomics models further supports the need for standardized ROI definition, robustness assessment, and calibration/decision-curve evaluation in glioma imaging studies (42–51).

Incorporating the RadScore into the clinical-imaging baseline improved the validation AUC from 0.744 to 0.811 and reduced the Brier score from 0.178 to 0.159. These findings support the complementary value of radiomic heterogeneity beyond conventional clinical and advanced imaging variables. The integrated model also provides a clinically interpretable multimodal framework by jointly considering radiomic, perfusion-diffusion, inflammatory, molecular, and treatment-related information. Given the modest validation sample size and single-center design, these comparative results should be interpreted cautiously and require external confirmation.

Several limitations should be acknowledged. First, although 13 patients classified as PsP underwent repeat surgery or biopsy with histopathological assessment, the endpoint for most patients was based on RANO/RANO 2.0-guided longitudinal clinical-radiological assessment rather than uniform tissue confirmation. To reduce misclassification, non-histologically confirmed PsP required at least 6 months of stable or improved follow-up imaging, outcome adjudication was performed through multidisciplinary review by two radiologists and two medical oncologists, and 21 indeterminate cases were excluded before model development. Second, the retrospective single-center design may introduce selection bias and limit generalizability. Third, although the leakage-controlled split-first workflow reduced the risk of information leakage, the validation cohort was relatively small and derived from the same institution; therefore, model calibration and comparative performance among the integrated, clinical-imaging, and RadScore-only models should be interpreted cautiously. Fourth, no independent external validation cohort was available; therefore, all performance estimates remain internally validated only. Fifth, complete-case analysis may introduce bias if excluded patients differed systematically from included patients. Sixth, scanner hardware, acquisition protocol, image preprocessing, ADC/rCBV measurement, and ROI delineation can affect quantitative imaging features; ICC filtering reduces segmentation-related instability but does not fully address acquisition-related heterogeneity. Prospective multicenter validation with standardized imaging protocols, locked radiomics code, complete raw feature matrices, and, where feasible, histopathological or rigorously adjudicated reference standards is required.

## Conclusion

In conclusion, this single-center exploratory study developed a leakage-controlled split-first prediction workflow for PsP after radiotherapy in patients with HGG. The nested RadScore retained validation-set discriminative information and modestly improved the clinical-imaging model when incorporated into an integrated multimodal framework. This model may support individualized PsP risk estimation by combining radiomic, perfusion-diffusion, inflammatory, molecular, and treatment-related information, but external validation with standardized imaging protocols and locked model coefficients is required before clinical implementation.

## Data Availability

The datasets used and analyzed during the current study are available from the corresponding author upon reasonable request, subject to patient privacy protection, institutional policies, and approval by the relevant ethics committee where required.
