# Supplementary material for: Radiomics and clinical data predict pseudoprogression after radiotherapy in high-grade glioma
Source: Front Oncol. 2026 Jul 20;16:1892760. doi: 10.3389/fonc.2026.1892760 (PMC13429429; doi:10.3389/fonc.2026.1892760)
Supplement: Supplementary file 1 [file DataSheet1.docx]

**Supplementary Table S1**

*Detailed acquisition parameters for conventional MRI and DSC-PWI perfusion imaging*

| **Imaging sequence** | **Sequence type** | **TR/TE (ms)** | **TI (ms) / flip angle** | **Matrix size** | **FOV (mm)** | **Slice thickness / gap (mm)** |
| --- | --- | --- | --- | --- | --- | --- |
| T1WI | Spin Echo / FSE | 2000 / 15 | - / 90 degrees | 256 x 256 | 230 x 230 | 5.0 / 1.0 |
| T2WI | Fast Spin Echo | 4000 / 100 | - / 90 degrees | 320 x 320 | 230 x 230 | 5.0 / 1.0 |
| T2-FLAIR | Fluid-attenuated IR | 8500 / 110 | TI: 2200 | 256 x 256 | 230 x 230 | 5.0 / 1.0 |
| DWI (ADC) | Single-shot EPI | 3500 / 75 | b = 0, 1000 s/mm2 | 128 x 128 | 240 x 240 | 5.0 / 1.0 |
| T1CE (3D) | 3D MPRAGE / BRAVO | 2300 / 2.8 | TI: 900 / FA: 9 degrees | 256 x 256 | 240 x 240 | 1.0 / 0.0 isotropic |
| DSC-PWI | Gradient-echo EPI | 1500 / 30 | FA: 60 degrees | 128 x 128 | 220 x 220 | 5.0 / 0.0 full brain |

DSC-PWI contrast protocol: 60 dynamic phases were acquired with a temporal resolution of approximately 1.5 seconds. A preload dose of 0.05 mmol/kg gadolinium-based contrast agent was administered 5 minutes before perfusion imaging. During DSC acquisition, a main bolus dose of 0.1 mmol/kg was injected at approximately the 10th phase using a power injector at 4.5 mL/s, followed by a 20-mL saline flush.

Leakage correction: post-processing was performed using Syngo.Via or GE AW workstations. Bidirectional T1 and T2* leakage correction based on the Boxerman-Schmainda model was applied to calculate corrected rCBV.
